# Superior sulcus non small cell lung carcinoma: retrospective analysis of 42 patients

**DOI:** 10.1186/s13014-014-0259-6

**Published:** 2014-11-26

**Authors:** Pierre Truntzer, Delphine N Antoni, Nicola Santelmo, Catherine Schumacher, Pierre-Emmanuel Falcoz, Elisabeth Quoix, Jean-Pierre Steib, Gilbert Massard, Georges Noël

**Affiliations:** Radiotherapy Department, Centre Paul Strauss, 3, rue de la Porte de l’Hôpital, BP 42, 67065 Strasbourg cedex, France; Radiobiology Laboratory EA 3430, Federation of Translational Medicine in Strasbourg (FMTS), Strasbourg University, Strasbourg, France; Thoracic surgery department, Nouvel Hôpital civil, 1, place de l’Hôpital, 67091 Strasbourg cedex, France; Pneumology department, Nouvel Hôpital Civil, 1, place de l’Hôpital, 67091 Strasbourg cedex, France; Orthopaedic Department, Hôpital Civil, 1, place de l’Hôpital, 67091 Strasbourg cedex, France

**Keywords:** Chemotherapy, Radiotherapy, Sulcus non-small cell cancer, Surgery, Survival

## Abstract

**Aims:**

Retrospective, monocentric analysis of localized superior sulcus non-small cell cancer (SS-NSCLC), article management.

**Materials and methods:**

Between 2000 and 2010, 42 patients have been treated for a SS-NSCLC. Median age was 54.7 years (34.5-86.8). Nineteen tumors (45.2%) were stage IIB, 18 were stage IIIA (42.9%) and 5 were stage IIIB (11.9%). Twenty-two patients were treated by pre-operative radiotherapy or chemoradiotherapy, 20 received exclusive radiotherapy or chemoradiotherapy. Preoperative and exclusive median radiotherapy doses were 46 Gy (40–47 Gy) and 51.8 Gy (40–70 Gy), respectively. All patients treated with chemotherapy received at least platinum. Mean follow up was 44.1 months (0–128 months).

**Results:**

Local, loco-regional and metastatic relapses occurred in 11 (26.2%), 2 (4.8%) and 15 patients (35.7%), respectively. Most common metastatic site was cerebral (7 patients, 46.7%). Median disease-free survival (DFS) was 9.7 months (8.9-10.4). One-, 2- and 5- years DFS rates were 44%, 33% and 26.5%, respectively. No prognostic factor was identified.

Median overall survival (OS) was 22.6 months (10.4-34.8). One-, 2- and 5- years OS rates were 61.9%, 44.9% and 30.1%, respectively. Univariate prognostic factors for OS were WHO (p = 0.027) and tumoral response (p = 0.05). In multivariate analysis, independent favorable prognostic factors were WHO 0–1 (p = 0.017; OR = 0.316 [CI95% 0.123-0.81) and complete response to treatment (p = 0.035; OR = 0.312 [IC95% 0.106-0.919]).

**Conclusion:**

This study highlighted that a good performans status and complete response to treatment are independent factors of OS, whatever the delivered treatment. Brain was the most common metastatic relapse site.

## Introduction

Superior sulcus non-small cell lung cancers (SS-NSCLC) represents less than 5% of the NSCLC [1]. In 1956, Chardack and Mc Callum reported the case of one patient treated with surgery followed by adjuvant radiation therapy delivering 65 Gy. This combined treatment allowed a long term survival without relapse at 70 months [2]. In 1961, Shaw and *al.* reported a retrospective study of 18 patients treated with pre-operative radiotherapy [3]. This combined treatment improved overall survival (OS), with 9 patients surviving at one year and a maximal disease-free survival (DFS) of 51 months. Thus, preoperative radiotherapy followed by surgery became the reference treatment for SS-NSCLC.

Pre-operative radiotherapy decreases the tumoral extension allowing a more complete resection, a lower local, lymphatic and systemic tumoral spread, and thereafter an improved local control. With a neoadjuvant radiotherapy, DFS and OS were increased by 30% and 15%, respectively, compared to surgery alone [4]. In order to improve these results, neo-adjuvant chemoradiation has been used in a phase II trial. In this trial, Fifty-seven patients among 76 (76%) underwent surgical resection, and pathologic complete resection was achieved in 51 patients (68%). There were 12 patients with pathologic complete response [5]. Thanks to these results, chemo-radiation followed by surgery became the cornerstone of treatment for SS-NSCLC [6]. For non operable patients with localized SS-NSCLC, a chemoradiation delivering at least 66 Gy in 2 Gy by fractions remains the standard of care [7].

In this retrospective institutional study, we report the outcomes of patients treated with different schedules.

## Material and methods

Between January 2000 and January 2010, 42 patients presenting a SS-NSCLC whose disease (4,2 patients/year/center) who met the study criteria were analyzed. Characteristics of the patients are summarized in Table 1. There were 11 women and 31 men (sex ratio 2,8). Mean age at diagnosis was 54.7 years. WHO performans status (WHO-PS) was 0–1 for 82.5% of the patients and 2 for 16.7% of the patients. Thirty-eight patients (90.5%) had shoulder pain irradiating to the scapula, whereas C8-D1 neuralgia, rib lyses and Claude Bernard syndrome were retrieved in 30 (71.4%), 20 (57.1%) and 4 patients, respectively. Only 3 patients had a complete Pancoast Tobias syndrome. These patients have been treated either by neo-adjuvant or exclusive radiotherapy with or without chemotherapy for IIB to IIIB SS-NSCLC SS-NSCLC.

**Table 1:** **Patient’s characteristics**

**Characteristics**	**# Patients**	**% Patients**
Gender		
Female	11	26.2%
Male	31	73.8%
Median age (min-max)	54.7 (34.5-86.8)	
WHO-PS		
0-1	33	82.5%
2	7	16.7%
NS	2	4.8%
Anatomopathology		
Adenocarcinoma	17	40.5%
Squamous cell carcinoma	14	33.3%
Large cell carcinoma	6	14.3%
Other	5	11.9%
Stage		
IIB	19	45.2%
IIIA	18	42.9%
IIIB	5	11.9%

NS: non specified.

### Pre-therapeutic assessment

The pre-therapeutic assessment included past medical story, clinical and biological exams and thoracic radiography. Non-invasive exams consisted for overall patients in a thoraco-abdominal and pelvic CT scan. Additional exams included a cervical and thoracic MRI in 25 patients (59.5%), a cerebral CT in 24 patients (57.1%), a PET CT in 24 patients (57.1%), a bone scintigraphy in 16 patients (38.1%) and a cerebral MRI in 4 patients (9.5%). Invasive exams consisted in a bronchoscopy for 38 patients (90.5%), a CT guided pulmonary biopsy for 30 patients (71.4%), a mediastinoscopy for 4 patients (10.8%). Staging was re-assessed according to the 7^th^ TNM classification. Functional respiratory evaluation was realized for 40 patients (97.6%), a gazometry for 27 patients (64.3%), a ventilation perfusion pulmonary scintigraphy for 15 patients (35.7%).

### Therapeutic management

Patients received either a preoperative chemoradiotherapy (RT-CT) or radiotherapy (RT) or an exclusive RT-CT or RT (Table 2).

**Table 2:** **treatment’s characteristic**

**Treatment**	**# Patients**	**% Patients**
Chemotherapy	36	85.7
# Mean cycle (min-max)	4 (2–6)	
Cisplatinum-vinorelbine	23	54.8
Carboplatin paclitaxel	10	23.8
Unknown	3	7.1%
Radiotherapy: total dose/dose per fraction		
66 Gy/2 Gy	8	19
46 Gy/2 Gy	22	52.4
Miscellaneous	12	28.6
Surgery	22	50%
*En bloc* resection	2	9.5
*En bloc* resection + lymph node dissection	20	90.5
Treatment		
Exclusive radiation treatment	3	7.1
Exclusive chemoradiotherapy	17	40.5
Radiation treatment + surgery	19	45.3
Chemoradiotherapy + surgery	3	7.1

#### Chemotherapy

Thirty-six patients received chemotherapy. Median delay between beginning of chemotherapy and radiotherapy was 5 weeks (0–18 weeks). Twenty-four patients received pre-radiation chemotherapy. One, 2, 3 or 4 chemotherapy cycles were delivered before start of radiotherapy in 12, 7, 3 and 2 patients, respectively. Seven patients received upfront chemoradiotherapy. This combination was followed by adjuvant chemotherapy for a total of 4 to 6 cycles. Two patients were treated with a sequential chemotherapy and radiation therapy. Two, 4, 10, 8 and 9 patients received 2, 3, 4, 5 and 6 cycles of chemotherapy, respectively. Overall patients received platinum based doublets, either combination of cisplatinun and vinorelbine (23 patients) or association of carboplatin and paclitaxel (10 patients). The details of chemotherapy were unknown for 3 patients.

#### Radiotherapy

Radiation treatment was delivered with 3D conformal radiotherapy (3D-CRT) by linear accelerator (Linac) for 38 patients (90.5%) or by intensity modulated radiotherapy (IMRT) with TomoTherapy HiArt® (Accuray Incorporated, Sunnyvale CA) for 4 patients (9.5%). 3D-CRT was delivered by two to four photons beams of 6–25 MV. The delineation of target volume and organs at risk was performed on a dosimetric CT scan (General Electric LightSpeed QX/i) with contrast injection and 3.75 mm thin joint images. For 4 patients, triple acquisition with blocked inspiration and expiration and free breathing was realized in order to define tumoral movement during the respiratory cycle [8]. For 8 patients a dosimetric TEP CT has been fused with the dosimetric CT scan.

GTV T (gross tumor volume) was delineated on the CT scan, CTV T (clinical target volume) was defined as the GTV T with an automatic 3D margin of 8 mm for adenocarcinomas, 6 mm for squamous cell carcinomas, and 5 mm for other subtype [9]. PTV (planning target volume) was CTV T with an automatic 3D 2 mm margin when triple acquisition was done, whereas a margin of 10 mm head-feet and 5 mm in other directions was added when no triple acquisition was performed. CTV N corresponded to the node area with lymph nodes which diameter reached 10 to 20 mm, presenting or not an increased uptake of ^18^FDG on the TEP CT, or lymph nodes smaller than 10 mm or greater than 20 mm but presenting an increased uptake of ^18^FDG. PTV N was defined adding a 5 mm margin around CTV N. Five patients underwent mediastinal invaded lymph nodes irradiation, 6 patients (treated before 2006) received prophylactic lymph node irradiation and one patient had both [10].

#### Surgery

Surgery was performed with a mean period of 9.5 weeks (6.8-12.9) after radiation therapy completion. It consisted in all cases in an *en bloc* resection lobectomy, with mediastinal lymph node dissection for 20 patients. When mediastinal lymph nodes dissection was performed, a mean of 15 lymph nodes (6–36) was removed.

### Follow up

The response to the treatment was considered complete (CR) if there was a radiological disappearance of the measurable disease or if ypT0N0 was obtained in the pathological report. Partial response (PR) was a regression of more than 50% to the base line of the sum of the perpendicular diameters of all the measurable disease, whereas progressive disease was defined as an improvement of more than 25% of this sum. Stable disease (SD) corresponded in the cases that did not meet these endpoints.

Post therapeutic follow-up consisted in a thoracic radiography, cerebral and thoracoabdominal and pelvis CT scan every 3 months the first year and more distanced the years after.

### Statistical analyses

Statistical test used were the chi^2^ or the Fischer exact test for qualitative parameters and variance analyses or T test for quantitative parameters. Overall survival (OS) and disease-free survival (DFS) were analyzed with the Kaplan Meyer method. OS was defined as the time between the end of the first treatment and the patient’s death or last news. DFS was the time between the end of the first treatment and the first relapse (local, regional or metastatic). Univariate analysis was realized to identify prognostic factors. Parameters considered as significant were included in a multifactorial analysis. Multifactorial analysis was performed with a Cox regression. Overall analyzes were done with IBM SPSS Statistics v20 software (IBM Inc., Armonk, NY, USA).

## Results

At time of diagnosis, 19 tumors were classified stage T3N0, IIB (45.2%), 18 tumors, stage IIIA (42.9%, 1 T3N1, 3 T2N2, 14 T4N0) and 5 tumors, stage IIIB (11,9%, 2 T3N3, 2 T4N2, 1 T4N3).

Pathological diagnosis was obtained by bronchoscopy in 19% and by CT guided biopsy in 71.4%. Pathology was adenocarcinoma in 17 cases (40.5%), squamous cell carcinoma in 14 cases (33.3%), large cells carcinoma in 6 cases (14.3%) and miscellaneous in 5 cases (11.9%).

### Treatment

Twenty-nine patients underwent neoadjuvant RT-CT (26 patients) or RT alone (3 patients) before surgery (Table 1). Chemotherapy consisted in a cisplatine-vinorelbine combination for 18 patients and carboplatine-paclitaxel in 6 cases, and was not mentioned for 2 patients. Median delivered irradiation dose was 46 Gy (40–47 Gy). Among these 29 patients, seven patients (24.1%) were not operated on, because of progressive disease (3 patients), tumors invading Adamkiewitz or great vessel tumoral (3 patients) and one patient had a large vertebral invasion. Only one patient received complement radiotherapy to reach a curative total dose of 66 Gy.

Thirteen patients received exclusive RT-CT (10 patients) or RT alone (3 patients). Chemotherapy was cisplatine vinorelbine for 5 patients, carboplatin paclitaxel for 4 patients, and was not mentioned for one patient. Median delivered irradiation dose was 51.8 Gy (40–70 Gy).

### Toxicities

Fifteen patients had chemotherapy related toxicities. Thirteen patients had hematoxicity (36.1%) complicated with febrile neutropenia in 6 patients (16.7%). Seven patients (19.4%) had grade 2 nausea and/or vomiting. One patient developed, platinum-related, hearing loss. Four patients died from infection during chemotherapy for relapse.

Nineteen patients (45.9%) developed radiation related side effects. Complications were grade 1–2 for 17 patients and grade 3 for 2 patients. Only for one patient the radiation treatment was definitively stopped at 42 Gy on the 46 Gy scheduled.

Thirteen patients (59%) had post-surgery complications. Complications were neuropathic pain (6 patients, 27.3%), respiratory distress (3 patients, 13.6%), lung infection (3 patients, 13.6%), brachial plexite (2 patients) and haemoragia in one patient. No patient died post operatively.

### Tumoral response

Surgery was complete (R0) for 19 patients (86.4%) and incomplete (R1) for 3 patients (13.6%) (Table 3). Surgical section was invaded on the pleura for one patient, on rib for another one and vertebral for the last one. Two patients presented lymph nodes invasion. For 18 patients, lymphadenectomy showed no invaded lymph node. For two patients the data was missing.

**Table 3:** **Overall treatment response**

**Treatment**	**CR**	**PR**	**SD**	**PD**	**Unknown**
Exclusive RT-CT	3/17 (18%)	5/17 (28%)	4/17 (24%)	3/17 (18%)	2/17 (12%)
Exclusive RT	0/3	0/3	2/3	1/3	0/3
RT-CT-surgery	7/19 (37%)	9/19 (47%)	2/19 (11%)	1/19 (5%)	0/3
RT-surgery	1/3	2/3	0/3	0/3	0/3
**Total**	**11/42 (26%)**	**16/42 (38%)**	**8/42 (19%)**	**5/42 (12%)**	**2/42 (5%)**

CR: complete response; PR: partial response; RT: radiation therapy; RT-CT: chemoradiotherapy concomitant SD: stable disease; PD: progressive disease.

Overall, complete, partial, stable, progressive or unknown responses were retrieved in 11 patients (26%), 16 patients (38%), 8 patients (19%), 5 patients (12%) and 2 patients (5%), respectively (Table 4). One patient died during the treatment from tumoral progression. Among operated patients, complete pathological response (pT0N0) was concluded in eight patients (36%) (Table 4).

**Table 4:** **Pathological response for patients operated on**

**Preoperative TNM**	**Post-operative TNM**	**CR**	**PR**	**SD**	**PD**
T3N0	pT0pN0	7			
	pT1pN0		3		
	pT2pN0		1		
	pT3pN0 (−25% greatest tumoral diameter)		3		
	pT3pN0			1	
	pT4pN0				1
T3N2	pT2pN2		1		
	pT3pN1			1	
T4N0	pT0pN0	1			
	pT3pN0		3		
Total		8	11	2	1

Mean follow up was 44.1 months (1.3-128). Thirty patients (71%) were dead. Twenty died of tumoral evolution.

### Overall survival (OS)

**Figure 1:**
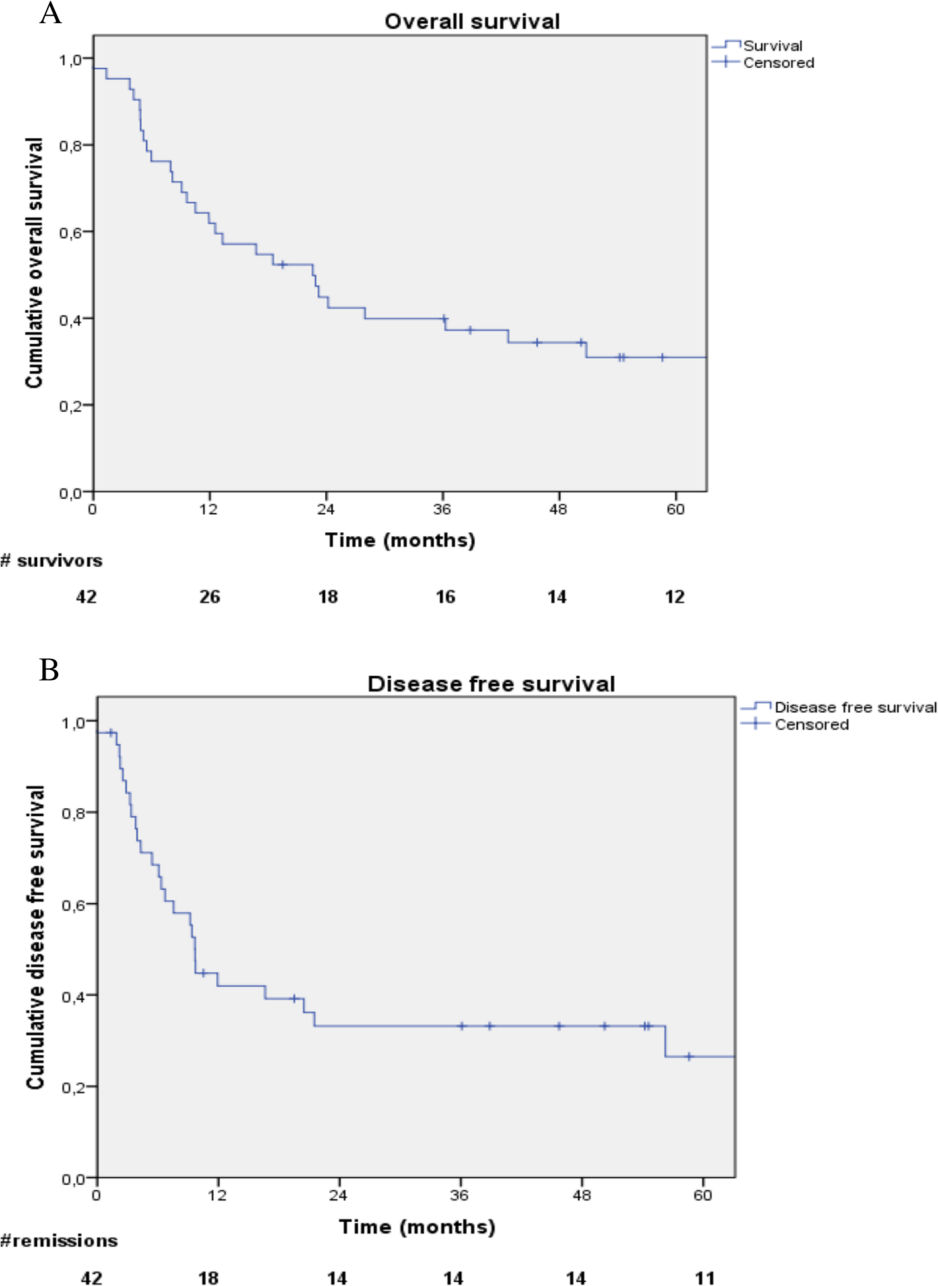
**Overall survival (A), disease free survival (B).**

**Figure 2:**
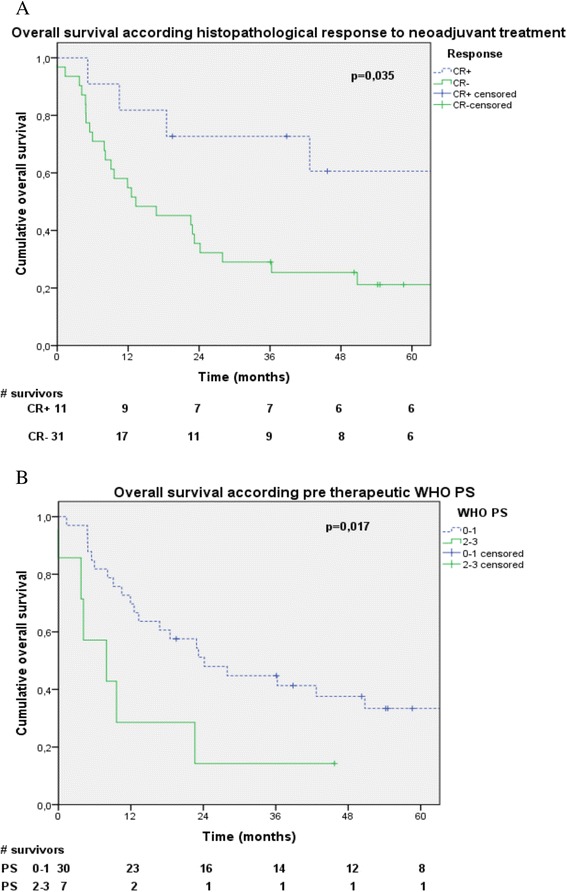
**Overall survival according neoadjuvant treatment response (A), according performans status at diagnosis (B).**

For all patients, median OS was 22.6 months (CI95% 10.4-34.8). One-, 2- and 5-years OS rates were 61.9%, 44.9% and 30.1%, respectively (Figure 1A). In univariate analysis significant differences have been retrieved for tumoral response (p = 0.046) and WHO PS (p = 0.027). In multivariate analysis, favorable OS prognostic factors were complete response after treatment (p = 0.035; OR = 0.312 [CI95% 0.106-0.919]) (Figure 2A) and WHO PS 0–1 (p = 0.071; OR = 0.316 [CI95% 0.123-0.817]) (Figure 2B).

Patients undergoing surgery had a median OS of 24 months (CI95% 0–48.7). One-, 2- and 5-years OS rates were 63.6%, 54.2% and 37.5%, respectively. No significant prognostic factor was identified in univariate or multivariate analysis.

**Figure 3:**
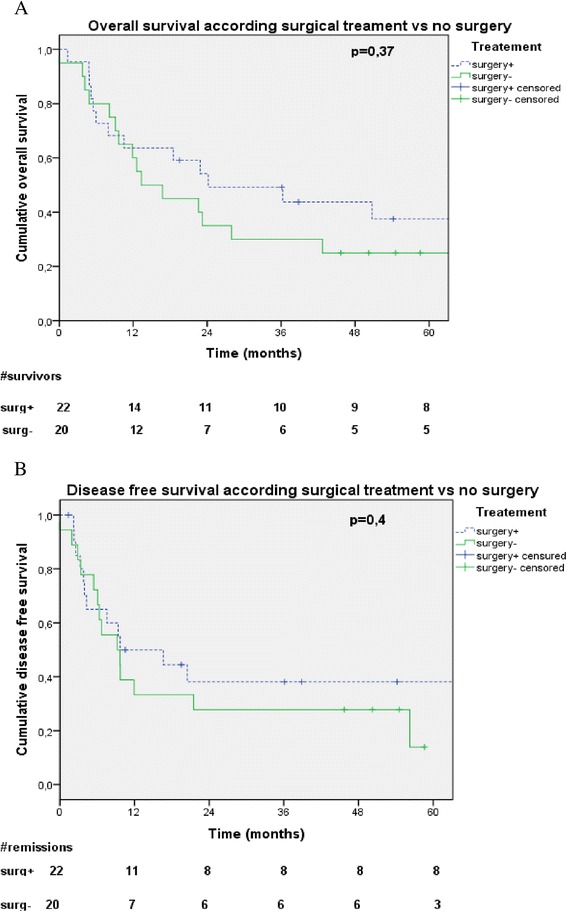
**Overall survival (A) and disease free survival (B) according to treatment with or without surgery.**

For patients who received exclusive RT-CT or RT, median OS was 13.3 months (CI95% 4.1-22.5). One-, 2- and 5-years OS rates were 60%, 35% and 25%, respectively. No significant prognostic factor was found in univariate or multivariate analysis. There was no significant difference in terms of OS between surgery or not (p = 0.37) (Figure 3A).

### Disease free survival (DFS)

Local relapse was diagnosed in 11 patients (26.2%), loco-regional in 2 patients (4.8%) and metastatic in 15 patients (35.7%). Most common metastatic relapse was the brain (7 patients, 46.7%). Median DFS was 9.7 months (CI95 8.9-10.4) (Figure 1B). One-, 2-, 3- years DFS rates were 44%, 33% and 26.5%, respectively. No significant prognostic factor was found in univariate or multivariate analysis.

For patients undergoing surgery, local relapse was retrieved in 3 of them (14.3%) and metastatic in 10 patients (45%). Most common metastatic relapse localization was brain (4 patients, 40%). Median DFS was 9.7 months (CI95% 0–24.8). One-, 2-, 3- years DFS rates were 50%, 38.1% and 38.1%, respectively. No prognostic factor was significant on univariate or multivariate analysis.

For patients undergoing exclusive RT/RTCT, local relapse was diagnosed in 8 patients (40%), loco regional in 2 patients (10%) and metastatic in 5 patients (25%). Most common metastatic relapse localization was brain (3 patients, 60%). Median DFS was 9.2 months (CI95% 4.48-13.9). One-, 2-, 3- years DFS rates were 33.3%, 27.8% and 13.9%, respectively. No prognostic factor was significant on univariate or multivariate analysis. There was no significant difference in terms of DFS between surgery or not (p = 0.4) (Figure 3B).

## Discussion

The treatment of SS-NSCLC is an exemplary multidisciplinary management. Since the 50’s, the recommendations have changed regularly. Since the incurability, with median OS between 3 and 14 months to an OS rates ranged between 18 and 33 months with preoperative chemoradiotherapy followed by *en bloc* resection for operable tumors. The incidence of this type of tumor with this localization is rare, the number of 4.2 patients/years in our study is similar to the principal studies [1,5,6,11-13]. Because of this low incidence, analysis of a large number of patients is biased by the heterogeneity of treatments along the large period needed to collect enough patients and data [1,5,6,11-13].

Two prospective studies have been published. The SWOG-9416 phase II trial included 110 patients with operable T3-T4 N0-1 SS-NSCLC and evaluated preoperative chemoradiotherapy delivering 45 Gy in 25 fractions of 1.8 Gy associated with 2 cycles combined cisplatine and etoposide [6,13]. The JCOG-9806 study included 75 patients presenting NSCLC and analyzed preoperative chemoradiotherapy delivering 45 Gy in split course (27 Gy in 15 fractions of 1.8 Gy, one week rest and 18 Gy in 10 fractions) associated with 2 cycles of mitomycine, vindesine and cisplatine [5].

The treatment completion rates were 76% in the both SWOG-9416 [6,13] and, JCOG-9806 [5] trials. In our study, 22 patients (75.9%) of the 29 planned for surgery, completed treatment. This ratio is favorably comparable to the prospective studies mentioned above.

In surgical cohorts, non-invaded surgical margins (R0) are the determining outcome factor concerning OS and DFS [1,12,14]. In the present study R0 margins rate was 86.3%, comparable to JCOG results (81.3%) [5], but lower than those obtained in the SWOG trial (96%). Patients deemed undergoing surgery have to be carefully selected and spine MRI may be essential to improve this selection.

In order to get a higher rate of complete response to neoadjuvant treatment, and to achieve more complete resections, radiation dose escalation has been evaluated. Kwong and *al.* reported retrospectively 36 patients, with stage IIB to IV with one resectable metastasis [15]. Chemotherapy was associated with conformal 3D radiation therapy at the dose of 45 Gy (25 fractions of 1.8 Gy) delivered in the tumor and mediastinum, with a complement of 14.2 Gy to the tumoral site. Complete resection was obtained in 100% of patients. In 2008, Kappers and *al.* reported 17 patients treated before surgery with 66 Gy in 24 fractions of 2.75 Gy and weekly concomitant cisplatine. R0 margins surgery was obtained for 12 patients [16]. Escalading the preoperative radiation dose, can improve R0 surgical margins rate and complete response to neoadjuvant treatment. However, this increasing of dose can lead in some complications. In our series, with classical doses, relapse rates after surgery remain low (14.3%) and are comparable with the rates obtained in the SWOG and JCOG cohorts: 23% and 13%, respectively [1,5,13].

Histological complete response could be a surrogate of overall survival. In the SWOG and JCOG trials, the rates were 33.7% and 16%, respectively [1,5,13]. In our study, 8 patients on 22 undergoing surgery (36%) had histological complete response. It seems that, the rate can be improved up to 40-47% by the increasing of the dose [15,16]. However, the benefit of dose escalation remains to be proved.

Median OS and Two- 3- and 5-years OS rates of our study were favorably comparable with those obtained in the SWOG and JCOG trials [5,6,13].

For patients with localized, but non-operable tumors (Stage IIIA with N2 or IIIB), the reference treatment remains chemoradiotherapy. Actually, the recommended dose is 66 Gy in 33 fractions of 2Gy. De Bari and *al.* evaluated, prospectively, 14 patients treated with chemoradiotherapy with a median radiation dose of 72 Gy (64–74) in 2 Gy fractions [17]. Median OS was 20 months. Two patients initially considered as non-operable, presented a partial response and have been operated on. Pathology did not find any tumor. No complication has been reported. The increase of dose for all the non-operable or limit of operability patients becomes allowed by the use of intensity modulated radiotherapy (IMRT) or proton therapy [18]. Limiting irradiated volumes, surgery could be feasible without increase of complication even if radiation dose has been increased. This schedule avoids split course schedule less efficient than continuous irradiation. This proposal still remains to be proved.

Some patients can be at the limit of the resection possibility at presentation. Neoadjuvant treatment could allow surgery. In absence of response, total radiation has to be increased to reach curative dose, and this with a short gap between the both irradiation periods. The evaluation of the response remains difficult. In SWOG study, 72% patient considered as PR at the radiological evaluation were indeed pathologically CR, and 65% considered as SD were CR or with few residual tumoral foci. The TEP CT could be helping but an improvement of this technic is needed to use it as an efficient exam in this indication [19].

Because of the risk of brain metastasis as first relapse pattern, a prophylactic brain irradiation can be discussed. However, RTOG trial concluded to a decrease of brain metastasis relapse with irradiation but did not show any overall survival advantage of this irradiation in advanced NSCLC and an increase of memory decline risk [20,21].

## Conclusion

Although our series is retrospective, number of patients and delivered treatments are comparable with previously published retrospective studies. Our results are highly comparable with those of prospective controlled studies. In addition, we have showed, that following closely the treatment guidelines, complete resection can be achieved and that complete pathology response is obtained in more than one quarter of the treated cases. We also reported a high risk of brain metastasis evolution in the patients with controlled disease. This observation highly questions about the prophylactic brain irradiation.

### Consent

Written informed consent was obtained from the patient for the publication of this report and any accompanying images.
